# Alpha-single chains of collagen type VI inhibit the fibrogenic effects of triple helical collagen VI in hepatic stellate cells

**DOI:** 10.1371/journal.pone.0254557

**Published:** 2021-09-02

**Authors:** Christian Freise, Hyunho Lee, Christopher Chronowski, Doug Chan, Jessica Cziomer, Martin Rühl, Tarkan Dagdelen, Maik Lösekann, Ulrike Erben, Andre Catic, Werner Tegge, Detlef Schuppan, Rajan Somasundaram, Ergun Sahin

**Affiliations:** 1 Department of Gastroenterology, Infectious Diseases and Rheumatology, Charité – Universitätsmedizin Berlin, corporate member of Freie Universität Berlin and Humboldt-Universität zu Berlin, Berlin, Germany; 2 Department of Radiology, Charité – Universitätsmedizin Berlin, corporate member of Freie Universität Berlin and Humboldt-Universität zu Berlin, Berlin, Germany; 3 Huffington Center On Aging, Baylor College of Medicine, Houston, Texas, United States of America; 4 Department of Cell Biology, Baylor College of Medicine, Houston, Texas, United States of America; 5 Department of Chemical Biology, Helmholtz Centre for Infection Research (HZI), Braunschweig, Germany; 6 Division of Gastroenterology and Hepatology, Beth Israel Deaconess Medical Center, Harvard Medical School, Boston, Massachusetts, United States of America; 7 Institute of Translational Immunology and Research Center for Immune Therapy, University Medical Center, Mainz, Germany; 8 Department of Emergency Medicine, Charité – Universitätsmedizin Berlin, corporate member of Freie Universität Berlin and Humboldt-Universität zu Berlin, Berlin, Germany; 9 Department of Physiology and Biophysics, Baylor College of Medicine, Houston, Texas, United States of America; University of Navarra School of Medicine and Center for Applied Medical Research (CIMA), SPAIN

## Abstract

The interaction of extracellular matrix (ECM) components with hepatic stellate cells (HSCs) is thought to perpetuate fibrosis by stimulating signaling pathways that drive HSC activation, survival and proliferation. Consequently, disrupting the interaction between ECM and HSCs is considered a therapeutical avenue although respective targets and underlying mechanisms remain to be established. Here we have interrogated the interaction between type VI collagen (CVI) and HSCs based on the observation that CVI is 10-fold upregulated during fibrosis, closely associates with HSCs in vivo and promotes cell proliferation and cell survival in cancer cell lines. We exposed primary rat HSCs and a rat hepatic stellate cell line (CFSC) to soluble CVI and determined the rate of proliferation, apoptosis and fibrogenesis in the absence of any additional growth factors. We find that CVI in nanomolar concentrations prevents serum starvation-induced apoptosis. This potent anti-apoptotic effect is accompanied by induction of proliferation and acquisition of a pronounced pro-fibrogenic phenotype characterized by increased α-smooth muscle actin, TGF-β, collagen type I and TIMP-1 expression and diminished proteolytic MMP-13 expression. The CVI-HSC interaction can be disrupted with the monomeric α2(VI) and α3(VI) chains and abrogates the activating CVI effects. Further, functional relevant α3(VI)—derived 30 amino acid peptides lead to near-complete inhibition of the CVI effect. In conclusion, CVI serves as a potent mitogen and activating factor for HSCs. The antagonistic effects of the CVI monomeric chains and peptides point to linear peptide sequences that prevent activation of CVI receptors which may allow a targeted antifibrotic therapy.

## Introduction

The differentiation of hepatic stellate cells (HSC) and portal fibroblasts into highly proliferative myofibroblasts upon chronic liver injury is considered a key pathogenic event in the development of liver fibrosis that ultimately can lead to cirrhosis and liver failure [1]. Myofibroblasts are the main extracellular matrix (ECM)-producing cells whose chronic activation results in the excess deposition of ECM components. Although the signaling events that drive myofibroblast formation and maintain their activated state are not completely understood, various studies have pointed to the importance of various growth factors, cytokines and extracellular components in these processes [2–4]. ECM components are implicated in myofibroblast regulation through several mechanisms including storage and release of cytokines and growth factors and direct ECM-cell interaction via cell surface receptors that activate growth factor signaling pathways in myofibroblasts [5–7].

Amongst the distinctly altered ECM components in human cirrhotic livers is collagen type VI (CVI) [8,9]. In comparison to other members of the collagen superfamily, CVI is characterized by several unusual properties including a complex gene structure, a multi-step intracellular assembly and distinct extracellular organization [10]. CVI is encoded by six different genes, COL6A1 to COL6A6. COL6A1, COL6A2 and COL6A3 encode the α1(VI), α2(VI), and α3(VI) chains that assemble at an equimolar ratio to form the CVI triple helix, which is expressed in different tissues [10–12]. In contrast, the expression of the recently identified α4(VI), α5(VI), and α6(VI) chains, which are structurally similar to the α3(VI) and can replace α3(VI), is more restricted [13,14]. The complex CVI maturation process involves a stepwise assembly of the three chains into triple-helix monomers that then form antiparallel dimers and tetramers [15,16]. After secretion into the extracellular space, tetramers assemble into a highly organizing microfibrillar network, which plays an important role in tissue organization by tethering cells to their environment through integrin and NG2 cell surface receptors [10]. CVI also interacts with several other ECM components including collagens (types I, IV and XIV), decorins and syndecans and thereby contributes to tissue organization. The importance of CVI for maintenance of cellular viability and tissue organization is underscored by studies in patients with CVI mutations who display increased apoptosis and tissue degeneration in proximal muscles and who develop various muscle disorders including Bethlem myopathy and Ulrich congenital muscular dystrophy [17,18]. Genetic evidence for a critical role of collagen type CVI in providing critical cell survival and proliferation signals comes also from mice deficient of CVI, which have increased apoptosis and blunted proliferation and regeneration in muscles [19]. These in vivo data are supported by cell culture studies demonstrating that triple-helical CVI prevents apoptosis and promotes proliferation through at least partially integrin-independent mechanisms suggesting that direct CVI-cell interactions have a profound effect of cell survival and proliferation [20,21].

In healthy livers, CVI is expressed in the interstitial ECM and is mainly distributed in the portal areas. During liver fibrosis CVI is highly expressed in myofibroblasts and can be found along fibrous septa and bridging septa of cirrhotic nodules [22–24]. Interestingly, the dynamic process of tissue remodeling characterized by ECM deposition and degradation is associated with release of soluble collagen CVI fragments. Such proteolytic CVI fragments are associated with fibrosis in animal models and cirrhosis in patients [25,26].

Here we have characterized the impact of CVI on survival, proliferation and activation in primary HSCs and report that the triple helical pepsin solubilized CVI is a survival and growth factor. We find that the α2(VI) and α3(VI) and α3(VI)-derived peptides can compete with CVI and inhibit CVI effects.

## Materials and methods

If not noted otherwise, chemicals and reagents were purchased from Sigma-Aldrich (Deisenhofen, Germany) or Merck (Darmstadt, Germany).

### Animal studies

All animal studies were conducted according to the European Guidelines for animal welfare (2010/63/EU) with approval of the commission for animal experiments headed by the “Landesamt für Gesundheit und Soziales” (LaGeSo, Berlin, Germany). Animal welfare was monitored twice daily by trained personnel.

### Isolation of collagens and collagen single chains

Human collagens I (CI) and VI (CVI) as well as the alpha collagen chains from CVI (α1-3(VI)) were isolated from placentas by pepsin digestion, fractional salt precipitation in acidic and neutral buffers, ion exchange and molecular sieve chromatography as described previously [8,20]. All preparations were tested for purity by SDS-PAGE and amino acid analysis after hydrolysis. Purified collagens were lyophilized and dissolved in 0.25 or 0.5 mol/l acetic acid (HAc) at a concentration of 2 mg/ml before use. Growth factor contamination of CVI was excluded by molecular sieve chromatography with a cut-off of 100 kDa in a buffer containing 6 mol/l guanidine and reconstitution in neutral guanidine-free buffers, or the use of neutralizing antibodies against certain growth factors as described before [20].

### Synthesis of α3(VI)-derived peptides

Solid-phase synthesis of the peptides was carried out on a scale of 25 μmole per peptide with a Syro Multiple Peptide Synthesizer (MultiSynTech, Witten, Germany) on Rapp S PHB resins (Rapp Polymere, Tübingen, Germany) with precoupled C-terminal amino acids. Fmoc chemistry with TBTU/diisopropylethyl amine activation with fivefold excess was employed. Coupling time was 1 h. Side chain protections of the amino acids were as follows: Asp, Glu, Ser, Thr and Tyr: t-Bu; Asn, Cys, Gln and His: Trt; Arg: Pbf; Lys and Trp: Boc. Peptides were cleaved from the resin and deprotected by a 3- hour treatment with TFA containing 3% triisopropylsilane and 2% water (10 ml/g resin). After precipitation with t-butylmethyl ether, the resulting crude peptides were purified by preparative HPLC (RP-18) with water/acetonitrile gradients containing 0.1% TFA and characterized by analytical HPLC and MALDI-MS. Final products were lyophilized from water. For the calculation of the concentration of stock solutions of the peptides for each basic amino acid (Arg, Lys and His) and for the free N-terminus one counter ion of trifluoroacetic acid (mol. weight 114.02) was taken into account. The amino acid sequences of the peptides are listed in S1 Table.

### Reactivation of CVI

Immediately before use, guanidine hydrochloride (6 mol/l) was cleared over night by activated charcoal (Merck, Darmstadt, Germany) and adjusted to pH 8.0 by adding 50 mmol/l tris(hydroxymethyl)aminomethane (Tris). Freeze dried CVI (10 mg/ml) was mixed with 1 ml Tris-buffered guanidine-hydrochloride, incubated at room temperature for 1 h with frequent thorough vortex followed by 1 h at 37°C. The buffer from the clear supernatant after centrifugation (10.000 x g, 10 min, 4°C) was replaced for 150 mmol/l HAc by gelfiltration (Sephadex-G25; GE Healthcare, Munich, Germany). This procedure regains the biological activity of CVI on CFSC (S1 Fig).

### Isolation and culture of primary rat hepatic stellate cells and CFSC cell culture

Primary HSC were obtained by in situ perfusion of livers of male Wistar rats (400–500 g, from a commercial breeder, Schönwalde, Germany) with Gey’s balanced salt solution (GBSS) containing 0.5 g/l collagenase P (Roche Molecular Biochemicals, Mannheim, Germany), 0.5 g/l Pronase (Merck, Darmstadt, Germany), and 7 mg/l DNase (Roche Molecular Biochemicals). The removed liver was digested with 100 ml GBSS containing 0.25 g/l collagenase, 0.2 g/l pronase and 7 mg/l DNase as described [27]. HSC were separated from other hepatic cells by density-gradient centrifugation on 11% nycodenz (Nyegaard, Oslo, Norway). The cells were then cultured in Dulbecco’s modified Eagle’s medium (Life Technologies, Inc., Paisley, Scotland) containing 20% fetal bovine serum (FBS), 100 U/ml penicillin, 100 mg/l streptomycin and 1 ml/l amphotericin B. After 24 h, the medium was changed to remove non-adherent cells and debris resulting in a >90% pure HSC-culture. The purity of cell preparations was assessed by their characteristic light-microscopic appearance and vitamin A autofluorescence [28]. Thereafter the medium was changed every 2–3 days. Cells were passaged at day 7–9 when they had acquired a myofibroblast-like phenotype. Cells of the third passage were used for all subsequent studies. The HSC cell line CFSC derived from cirrhotic liver of rats after treatment with carbon tetrachloride were obtained from W. Dieterich (Erlangen, Germany). As for maintenance, cells were cultured in standard medium consisting of Dulbecco’s Modified Eagle’s medium (DMEM) supplemented with 2 mmol/l L-glutamine, 1 mmol/l sodium pyruvate, 10^3^ U/ml penicillin, 10^3^ μg/ml streptomycin, 25 μg/l L-ascorbic acid and 10% FBS (10% FBS) (all from Life Technologies, Carlsbad, CA). Twice weekly, cells were detached by trypsin (0.05%) and ethylenediaminetetraacetic acid (EDTA; 0.02%; Biochrom, Berlin Germany) and new cultures were set up with 2x10^5^ cells/75 cm^2^ in 10% FBS. CFSC cultures were negative for *Mycoplasma spec*. as routinely assessed by specific polymerase chain reaction from the cell culture supernatants (VenorGEM; Biochrom, Berlin, Germany).

### Studies on effects of collagens on morphology and survival of primary hepatic stellate cells using phase contrast microscopy

Activated HSC (third passage) were serum starved for 24 h in standard medium without FBS (starving medium), followed by dissociation with 500 μl 0.05% trypsin-EDTA (Biochrom, Berlin, Germany) and neutralization with 30 ml starving medium containing 1 g/l soybean trypsin inhibitor and 1 g/l ovalbumin. Alternatively, cells were scraped carefully with a cell-scraper (Costar, Cambridge, MA, USA) to exclude any potential effect of trypsin. After centrifugation at 800 x g for 10 min, cells were washed twice with 20 ml PBS (w/o Ca^2+^, Mg^2+^; Biochrom, Berlin, Germany), resuspended in starving medium and plated at a density of 1.5-2x10^6^ in 1.7 ml starving medium in 6-well plates (NUNC, Wiesbaden, Germany). Cells were then treated with starving medium, soluble CI or CVI (both at a final concentration of 30 mg/l), or 10% FBS. For quantification of spreading, cells that had attached were counted 12 hours after plating and spreading efficiency was determined as percentage of plated cells in each group. De-novo DNA synthesis and apoptosis were assessed by morphological criteria, Cell death detection ELISA and FACS analysis as described below. Cells with or without addition of collagens cells were photographed either 24 h or five days after plating using a Zeiss phase contrast microscope at a 40x magnification. For immunostaining experiments, freshly isolated HSC were plated at a density of 5x10^5^ HSC in Lab-Tek^™^ chamber-slides. After 24 h, the cells were treated in standard medium containing 10% FBS with or without the presence of 30 mg/l CVI. After for 3, 7 or 14 days, the cells were washed with PBS and fixed for 10 min with methanol. After blocking the cells 1h with DMEM+1% FBS, the cells were incubated with primary antibodies against *a*-actin (Sigma-Aldrich, Taufkirchen, Germany, #A2547; dilution 1:200) and GFAP (Sigma-Aldrich, #G9269; dilution 1:20) followed by a washing step with PBS and the incubation with fluorescence-labeled secondary antibodies (Alexa Fluor 546 & Alexa Fluor 488, Molecular Probes, Eugene, USA, #A11030 & #A11034; dilution 1:100). Finally, cell nuclei were stained with 1 μg/ml DAPI (Sigma-Aldrich) in methanol. Cells were analyzed by fluorescence microscopy (Zeiss, Jena, Germany; Kodak Ektachrome P1600 and a quantitative staining analysis was performed using Image J software (version 1.50i; National Institutes of Health, Bethesda, MA, USA).

### Morphological studies on primary hepatic stellate cells by electron microscopy

5x10^5^ freshly isolated primary rat were cultured on Millicell-PCF 0.4 μm filters and treated with CVI or standard medium containing 10% FBS. After 4d or 12d the cells were fixed with glutaraldehyde (pH 7.2), buffered with cacodylic acid (pH 7.4, containing 4% sucrose) and fixed again with an aqueous 2% osmiumtetroxide solution. Afterwards, the cells were dehydrated in ethanol and were mounted in araldite. The sample blocks were then cut into very thin cross-sections, stained with uranylacetate and leadcitrate and analyzed by electron microscopy (Zeiss EM 10, Jena, Germany). Photos were taken using an ISO Kodak monochrome film (8.2 x 10.5 cm) and a magnification of 4.400. For documentation, the pictures were scanned using an AGFA Studio Scan IIsi device.

### Cell proliferation assays

5x10^3^CFSC in 100 μl 10% FBS were seeded into a 96-well flat-bottom tissue culture plate (Nunc, Roskilde, Denmark). After 24 hours, medium was completely removed, and cells were rinsed with 200 μl phosphate-buffered saline and 100 μl standard medium containing only 0.25% FBS were added. Cells were cultured for additional 24 h prior to treatment. Synchronized microcultures in 96-well plates were treated for 2 hours in reduction medium. When appropriate, the reduction medium was supplemented with 40 mg/l of α1(VI), α2(VI) or α3(VI). Then, 30 mg/l CVI or CI as control were added and cells were incubated for 24 h after which [^3^H]-thymidine was added for additional 24 h. To assess effects of α3(VI)-derived 30-mer peptides on CVI-induced proliferation of CFSC, the reduction medium was supplemented with a 100-fold molar excess of the respective 30-mer peptides compared to CVI. [^3^H]-thymidine incorporation was measured as described before [20]. Cells treated with 150 mmol/l HAc or grown in 10% FBS served as negative and positive controls, respectively. During the last 4 hours of treatment, all cultures received 18.5 kBq [^3^H]-thymidine (Amersham, Freiburg, Germany). Cells were fixed with 10% trichloroacetic acid and lysed by 200 mmol/l NaOH. After neutralization with 800 mmol/l HCl, lysates were collected to a glassfibre mat and the *de-novo* DNA synthesis was assessed by liquid scintillation (LKB Wallac, Bromma, Sweden) and the radioactive decay was counted (for) a minute (cpm).

### Gene expression analysis

5×10^4^ CFSC were plated in 6-well microplates in 3 ml 10% FBS and were incubated for 24 h before replacing the medium with standard medium containing 0.25% FBS. 30 mg/l CVI, 133 mg/l of the monomeric alpha chains of CVI and/or TGF-β (2 ng/ml) were added after 24 h. After 24 h incubation, total RNA was directly extracted from cell layers using the RNApure reagent (Peqlab Biotechnologie, Erlangen, Germany). Reverse transcribed complementary DNA templates were amplified by quantitative real-time polymerase chain reaction (PCR; Roche Diagnostics, Mannheim, Germany) using fluorogenic probes labeled with 6-carboxy-fluorescein and 6-carboxy-tetramethylrhodamin (Sigma- Genosys, Steinheim, Germany) and primer pairs (Invitrogen, Karlsruhe, Germany) specific for fibrosis-associated genes (for sequences see S2 Table). Gene expressions were normalized to glyceraldehyde phosphate dehydrogenase (GAPDH).

### Quantification of apoptosis

#### Fluorescence activated cell sorting (FACS) analysis

10^5^ of freshly set cells were treated with starving medium, CI, CVI or 10% FBS for 24 or 48 hours as described before. After trypsinization, equal cell numbers were washed with 10 ml PBS, fixed in 70% ethanol for 30 min at -20°C and suspended in 500 μl PBS, containing RNase A (250 mg/l) for 30 min at 37°C. Fixed cells were stained with propidium iodide (50 mg/l) in a final volume of 1 ml before being subjected to fluorescence activated cell sorting (FACS) analysis.

#### Cell Death Detection ELISA

10^5^ cells were seeded in 6-well plates and treated with starving medium or medium plus CI, CVI or 10% FBS. After 24 or 48 h floating and adherent cells were pooled, and lysed with 500 μl of 1% SDS, 10 mM Tris, pH 7.4 (lysis buffer), for 30 min at 4°C. Lysed cells were transferred to 1.5 ml Eppendorf tubes and centrifuged at 13,000 rpm for 20 min, to separate low molecular weight DNA (oligonucleosome-sized fragments derived from apoptotic cells) from high molecular weight DNA (from viable cells). 400 μl of the supernatant containing oligonucleosomes were transferred to another Eppendorf tube and diluted 1:5 with lysis buffer. 100 μl aliquots were used for the Cell Death Detection ELISA, which was performed according to the manufacturer’s instructions (Boehringer Mannheim, Germany). In brief, antibodies against histones were coated onto 96-wells, 100 μl lysate-samples containing oligonucleotides and histones were added and incubated for one hour. After washing, wells were incubated with anti-double strand DNA antibody coupled to peroxidase for one hour. After reaction with peroxidase substrate, absorbance was measured at 405 nm using an ELISA reader. Background values (lysis-buffer alone) were subtracted, and results are shown as enrichment factor [enrichment factor: OD (optical density) of the sample (apoptotic cells) divided by the OD of the corresponding control (viable cells, 10% FBS-group). A defined positive control was prepared using a hypertonic buffer, containing 10 mM Tris, 400 mM NaCl, 5 mM CaCl2 and 10 mM MgCl2, instead of starving medium.

#### DNA laddering

Cells were treated 24 h with 0% FBS, CI, CVI or 10% FBS. Equal cell numbers (1.5x10^6^) were used to detect oligonucleosomes using the Apoptotic DNA Ladder Kit (Boehringer Mannheim, Germany) according to the manufacturer’s instructions. The isolated DNA from each group was electrophoresed on a 1.5% Agarose-Gel at 75 V for 1.5 hours, stained with ethidium bromide and visualized via an UV light source (302 nm).

### Western-blot analysis

Dislodged cells were centrifuged at 800 x g/10 min and lysed with 100 μl lysis buffer. The protein concentration was determined using NanoOrange^®^ Protein Quantitation Kit (Life Technologies) according to the manufacturer’s instructions. Briefly, protein solutions were mixed with the diluted NanoOrange reagent in 1.5 ml polypropylene tubes and heated at 95°C for 10 min in a thermomixer (Peqlab, Erlangen, Germany). Tubes were centrifuged and 200 μl of each sample were transferred to a black 96 well flat-bottom plate (Greiner BioOne, Frickenhausen, Germany). The emitted fluorescence was assessed at 570 nm in a SpectraMax Gemini EM (Molecular Devices, Biberach an der Riß, Germany). Protein concentrations were calculated from a bovine serum albumin standard curve. Equivalent amounts (10–50 μg) of protein were used in 20 μl. Sample buffer (1:1) was added and the aliquots (20 μl final volume) were boiled for 5 min and subjected to 12% SDS-PAGE. After blocking for 1 h at room temperature in 10 mmol Tris-HCl, pH 7.5, 100 mmol NaCl, containing 0.1% Tween 20 and 3% bovine serum albumine (BSA), nitrocellulose-membranes were incubated either with polyclonal rabbit anti-rat antibodies to Bax (1:1000, sc-493), Bcl-2 (1:1000, sc783, both Santa Cruz, Santa Cruz, USA), α-actin (1:4000, A2547, Sigma) or β-actin (1:10,000, A5441, Sigma). Membranes were then washed in 10 mmol Tris-HCl, 100 mmol NaCl, 0.1% Tween 20, pH 7.5, and incubated with anti-rabbit IgG coupled to peroxidase (1:1000, A4416, Sigma) for 1 h at room temperature or directly developed (RC20H). Bands were detected with the ECL chemiluminescence reagent (Amersham-Pharmacia, Freiburg, Germany). After stripping in 0.1 M glycin-HCl, pH 2, for 20 min at room temperature, membranes were reprobed with appropriate primary and secondary antibodies. Band intensities were analyzed by densitometry and normalized to β-actin.

### Statistical analysis

Data were analyzed using Graphpad Prism (Graphpad, La Jolla, CA). Statistical differences were calculated by one-way or two-way ANOVA analysis (one-tailed) and Tukey’s multiple comparisons test. Statistics for S2 Fig were calculated by two-way ANOVA and Sidak’s multiple comparisons test. Differences p<0.05 were considered significant.

## Results

### CVI alone is sufficient to promote spreading, survival and proliferation of primary rat HSCs

**Fig 1:**
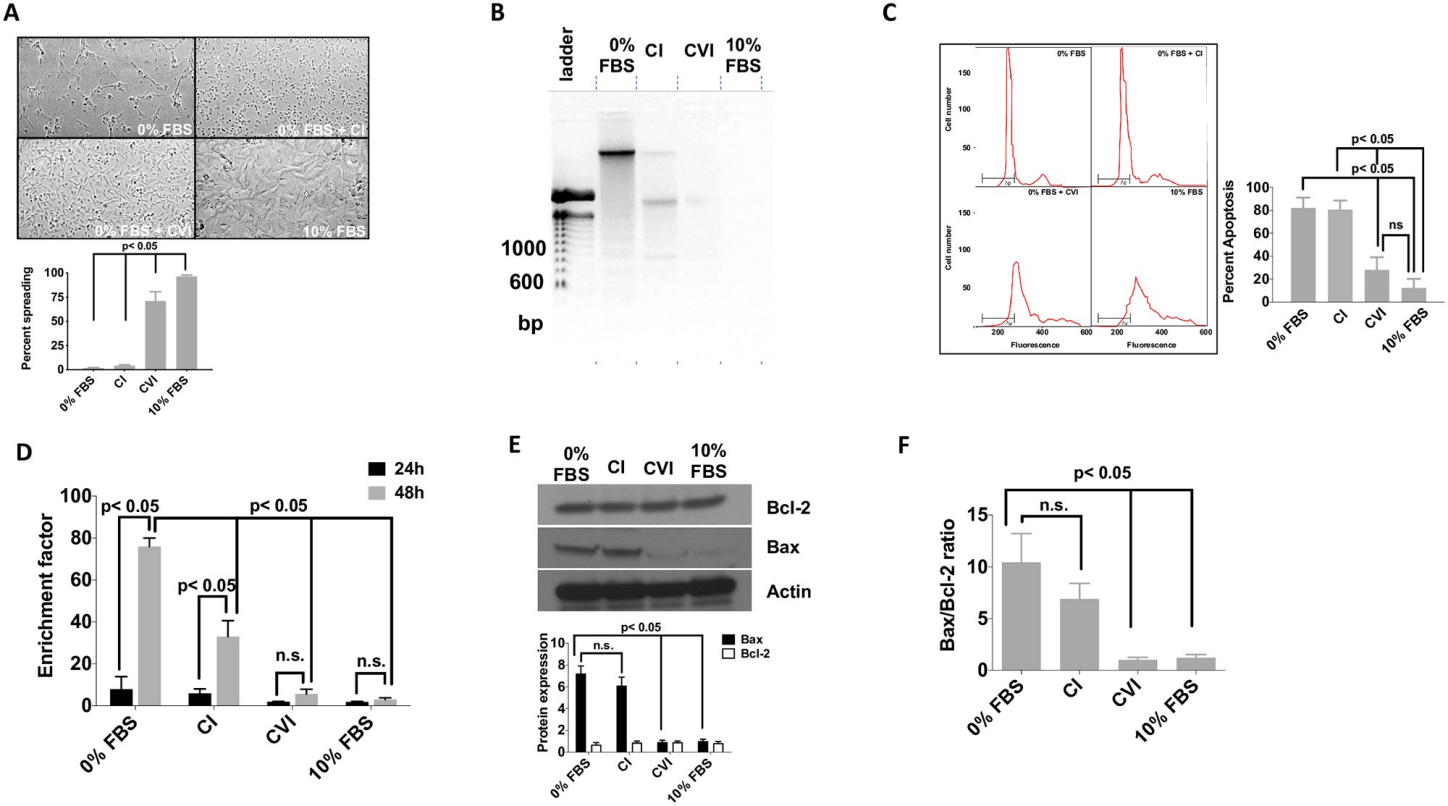
Soluble CVI induces HSC spreading and prevents apoptosis. (**A**) Primary rat HSC from the third passage were cultured 24 h in 6-well plates in serum-free medium, standard medium containing 10% FBS, or in serum-free medium supplemented with 30 μg/ml CI and 30 μg/ml CVI. Shown are representative images at 40-fold magnification from one out of three independent experiments. Graph below shows the quantification of spreading from three independent experiments. (**B**) 10^6^ serum-starved primary rat HSC were cultured for 24 h in medium alone, CI, CVI or 10% FBS. DNA was extracted and subjected to agarose gel electrophoresis to detect apoptosis-associated DNA laddering. Shown is a representative experiment out of three experiments. (**C**) Serum-starved activated HSC were grown in serum-free medium, CI, CVI or 10% FBS for 48 h. After fixation in 70% ethanol and RNase digestion, cellular DNA was stained with propidium iodide and subjected to FACS analysis. The integrated area of cellular DNA content was calculated as percentage of apoptotic cells. Right graph depicts quantification of apoptosis in each group from three independent experiments. (**D**) HSC were cultured for 24 or 48 hours and analyzed for the abundance low molecular weight oligonucleosomal DNA indicative of apoptosis by a sandwich ELISA. Shown are results of three independent experiments. (**E&F**) Expression levels of Bax and Bcl-2 was determined in activated HSCs by western blotting after 24 hours cultured in 0% FBS alone or in the presence of CVI and CI or 10% FBS. Shown is a representative blot from three independent experiments; densitometric analysis is derived from three independent experiments and ratio of Bax to Bcl-2 is shown. Statistics were calculated by one-way ANOVA analysis and Tukey’s multiple comparisons test. Differences p<0.05 were considered significant.

To test the effect of soluble CVI on spreading and survival of HSCs, we treated primary rat HSCs (rHSCs) immediately after isolation with either CVI, collagen type I (CI), collagen type IV (CIV) or fibronectin in medium containing or lacking 10% fetal bovine serum (FBS). While cells grown in 10% FBS medium adhered and viable while rHSCs grown in medium devoid of FBS showed significant less attachment and signs of cell shrinkage pointing to enhanced cell damage within 24 h (Fig 1A, representative image of three independent experiments). Strikingly, this growth factor withdrawal-induced apoptosis was potently suppressed by the addition of CVI and to a distinctly weaker extent by CI, which served as a control.

We subsequently investigated effects of CVI on apoptosis. Primary rHSCS were grown in medium alone or supplemented with either soluble CVI, CI, or 10% FBS. Apoptosis was assessed by electrophoretic, FACS and ELISA-based oligonucleosomal DNA fragmentation analysis. The typical apoptosis–associated DNA laddering, which indicates significant apoptosis within a cell population, was readily detected after 24 h in cells grown in medium alone and to a weaker extent in the presence of CI, whereas only faint or no DNA fragmentation was visible in cells treated with 30 μg/ml CVI or 10% FBS, respectively (Fig 1B, three independent experiments). A parallel FACS analysis revealed that 80% of primary rHCS grown in medium or CI underwent apoptosis within 24 hours, which was significantly inhibited by CVI or 10% FBS (25% and 12% apoptosis respectively). Compared to 80% apoptotic cells in the CI-treated group, the CVI-treated group had approximately 3-fold less apoptosis (25%) (Fig 1C; three independent experiments, p<0.05). Finally, the generation of oligonucleosomal fragments formed during apoptosis increased significantly in the medium alone over a 48-hour observation period (the enrichment factor rose from 8 after 24 h to 70 after 48 h). In contrast, CVI potently reduced the generation of oligonucleosomal fragments, and this was significantly stronger compared to CI based on the lower enrichment factor (for CVI this was 2 and 6 versus 5 and 30 in the CI-treated group after 24 and 48 hours, respectively; Fig 1D; three independent experiments, p<0.05).

To understand the molecular basis of this CVI-dependent anti-apoptotic effect we analyzed protein levels of two key opposing apoptosis regulators, the pro-apoptotic Bax and anti-apoptotic Bcl-2 proteins, which had been implicated previously in CVI-mediated effects in cancer cell lines [29,30]. Western blot analysis showed that rHSCs subjected to serum starvation or treated with CI up-regulated Bax without changing Bcl-2 protein levels (Fig 1E; representative blot from three independent experiments). In contrast, the addition of CVI was sufficient to suppress Bax up-regulation following serum starvation. Quantification of Bax protein levels showed similar Bax expression levels in the 10% FBS and CVI-treated group, which was 6-7-fold lower when compared to the medium or CI group (Fig 1E; three independent experiments; p<0.05). The Bax to Bcl-2 ratio as an indicator of overall apoptosis in a cell population was significantly lower in the CVI and 10% FBS group (1.0 and 1.2, respectively) compared to medium and CI group (10.4 and 7.0; Fig 1E; three independent experiments; p<0.05).

**Fig 2:**
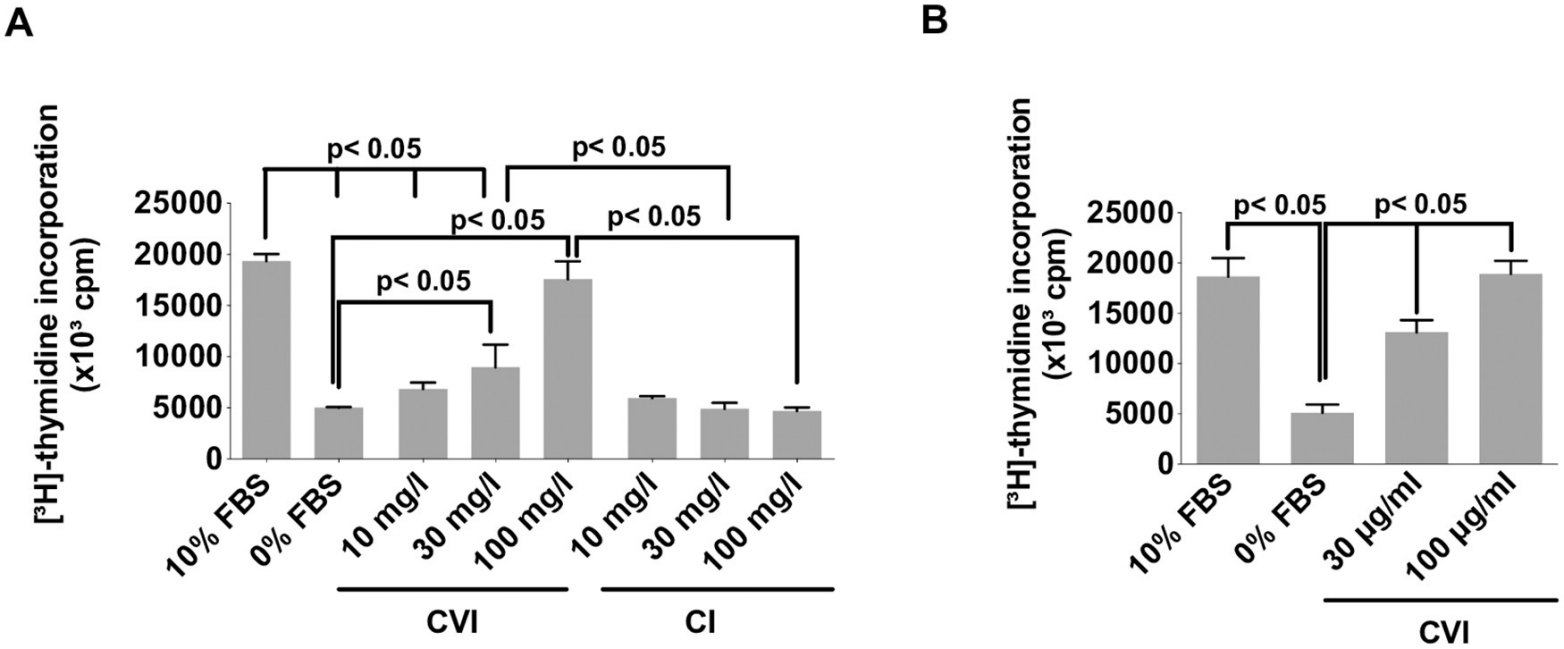
Soluble CVI is sufficient to increase HSC proliferation. (**A**) Serum-starved primary HSCs were treated with increasing concentrations of CI, CVI or 10% FBS as a positive control for 24 h. Proliferation was determined by [^3^H]-thymidine incorporation. Shown is the Mean ± SD from three independent experiments(**B**) CFSCs were incubated with indicated concentrations CVI for 24 h and proliferation was assessed by [^3^H]-thymidine incorporation. Cultures receiving equivalent amounts of the CVI solvent acetic acid (HAc) alone served as negative controls. Mean values ± SD (n = 3). Differences were calculated by one-way ANOVA analysis and Tukey’s multiple comparisons test. Differences p<0.05 were considered significant.

To determine whether CVI also stimulates proliferation we determined de-novo DNA synthesis in rHSCs via [^3^H]-thymidine incorporation assay. While cells grown in medium alone or in the presence of CI showed little DNA incorporation, addition of CVI at a concentration of 10 μg/ml stimulated a robust de-novo DNA synthesis within 24 hours (Fig 2; three independent experiments; p<0.05). This effect was concentration dependent as increasing the CVI concentration from 10 μg/ml up to 100 μg/ml induced a gradual increase in DNA synthesis, equaling the effects of 10% FBS, the most potent proliferation stimulus known (Fig 2A; n = three independent experiments; p<0.05). This proliferation-stimulating effect of CVI was not restricted to primary rHSCs as the addition of CVI induced a comparable increase in de-novo DNA synthesis in an established rat HSC cell line, CFSC, (Fig 2B; n = three independent experiments; p<0.05).

### CVI stimulates the activation of HSCs and induces fibrosis-associated gene expression changes

**Fig 3:**
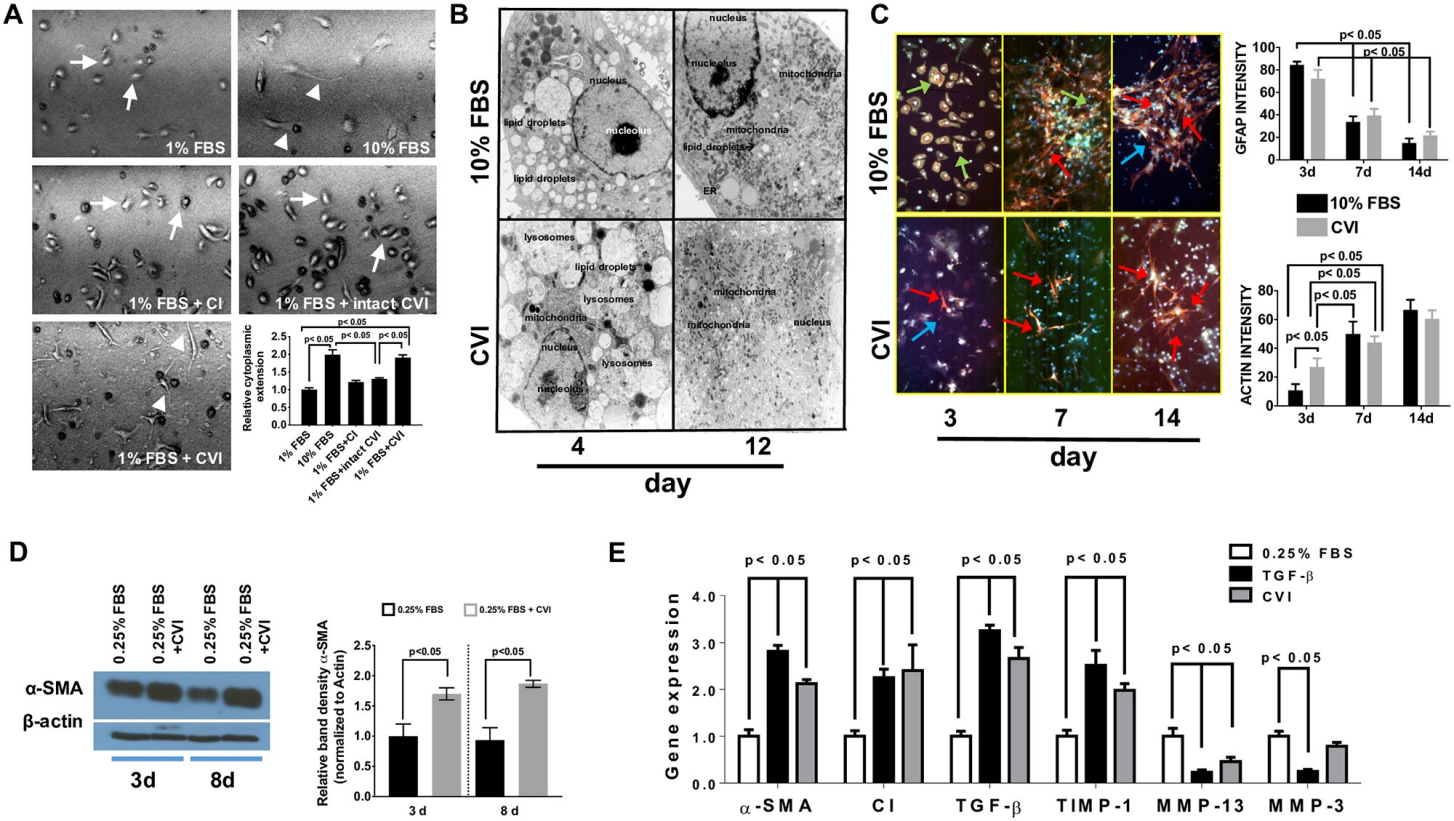
Effects of soluble CVI on activation and fibrosis-associated gene expressions in HSC. (**A**) Primary rat HSCs were plated at a density of 5x10^5^ cells in Lab-Tek^™^chamber-slides. After 24 h, cells were treated for five days with CI, CVI or with medium containing only 1% FBS or 10% FBS. 24 h after plating, cells were photographed using a Zeiss phase contrast microscope at 40x magnification; round-shaped arrows point to HSCs, arrow heads to cytoplasmic extensions. Cytoplasmatic extension was quantified from three independent experiments (lower graph) and expressed as Mean ± SD (**B**) Representative electron micrographs of freshly isolated rat HSCs treated either 4 or 12 days with medium containing 10% FBS with or without supplementation of soluble CVI (magnification: 4400-fold). (**C**) Freshly isolated rat HSCs were treated 3–14 days with medium containing 10% FBS with or without supplementation with soluble CVI. Gfap appears green (green arrows), α-Actin in red (red arrows). Cell nuclei were stained blue with DAPI (blue arrows). Shown are representative pictures from one out of three independent experiments and quantification of mean intensity for Actin and Gfap is shown on the right. (**D**) Expression levels of αSMA in rat HSCs was determined by western-blot analysis and normalized to actin. Shown is one western blot result from one out of three independent experiments (left) and blotted as relative expression of αSMA (right). (**E**) Effects of soluble CVI on gene expressions were determined by quantitative PCR and normalized to GAPDH. Serum-starved CFSC were treated as indicated for 24 h prior to the determination of gene transcriptions. Shown is Mean ± SD (n = 4). Statistics were calculated by one-way ANOVA analysis (3A, 3D, 3E) or two-way ANOVA (3C) and Tukey’s multiple comparisons test. Differences p<0.05 were considered significant.

A hallmark of HSCs is their ability to adopt an activated cellular phenotype that is characterized by a transdifferentiation process from a quiescent phenotype into a highly proliferative and metabolically active cell type that produces the majority of the ECM components. This activation includes morphological changes such as cytoplasmatic extension and loss of intracellular stored retinoids, and complex gene expression changes [31]. To study effects of CVI on morphological features of primary rat HSC, HSCs were treated with soluble CI, intact CVI and soluble CVI in serum-reduced medium containing 1% FBS to ensure survival of cells. In the absence of collagens, HSCs appeared small and showed only few cytoplasmic extensions indicating that cells remained predominantly quiescent under these conditions. While the addition of CVI and CI slightly increased cell attachment and cytoplasmic extensions, soluble CVI induced the formation of distinct cytoplasmic extensions to a degree similar to cells cultured in 10% FBS (Fig 3A; n = three independent experiments). These data were underlined by spreading experiments with primary rat HSC and treatment with different collagens (S2 Fig). Electron microscopy-based analysis further demonstrated that soluble CVI-treated cells underwent activation over a 12-day period as indicted by changes in lipid droplet and mitochondrial content [32]. While primary rHSCs contained abundant lipid droplets and only few mitochondria a few days after their isolation, soluble CVI-treated cells rapidly lost lipid droplets and showed increased mitochondrial content, both indicating activation of rHSCs (Fig 3B; n = three independent experiments) [32]. To further corroborate the CVI-induced activation, we determined the expression levels of glial fibrillary acidic protein (GFAP) and α- smooth muscle actin (αSMA), which represent markers for quiescent and activated HSCs, respectively. Time course analysis expression by immunohistochemistry showed that the expression of the quiescent marker GFAP declined from day 3 to day 14 while the expression of activation marker (αSMA) increased from day 7 to day 14 in the 10% FBS group. Interestingly, CVI strongly induced the protein expression of αSMA and this effect was rapid as demonstrated by the early expression of αSMA, which could be detected as early as three days post-stimulation by immunostaining (Fig 3C). This CVI-driven expression of αSMA was also evident when αSMA expression was quantified by immunoblotting (Fig 3D). We next determined the effects of CVI on the expression of fibrosis-associated genes in CFSC cells. TGF-β served as a positive control due to its well-established role in driving the expression of fibrosis-relevant genes such as αSMA, CI and TGF-β itself [31,32]. In contrast to medium alone and CI, CVI potently induced αSMA, CI and TGF-β expression (Fig 3E; n = 3; p< 0.05). Furthermore, in line with its activating effect, CVI stimulated the expression of TIMP-1 (tissue inhibitor of metalloproteinase 1, a critical inhibitor of ECM-degradation), while the expression of the matrix-degrading metalloproteinase MMP-13 was decreased. Importantly, these CVI-induced gene expression changes were comparable to changes observed with TGF-β stimulation (Fig 3E; n = 3; p<0.05). Some differences between CVI and TGF-1 were notable as CVI only slightly reduced the expression of MMP-3 whereas TGF-β potently reduced the expression of MMP-3 by ~70% indicating that not all MMPs were affected to the same degree by CVI (Fig 3E; p = 0.3012 and p<0.05, respectively). To compare the effects of CVI and TGF-β in more detail, we analyzed the time—dependent changes of CI, the most abundant collagen produced during fibrosis. TGF-β-induced CI expression increased after ~8h of treatment and reached a maximum after ~24h. The effects of CVI followed similar kinetics, although the response was slightly decreased and delayed (S3 Fig).

### The α3(VI) single chain and α3(VI)-derived peptides specifically inhibit CVI-induced HSC proliferation and activation

**Fig 4:**
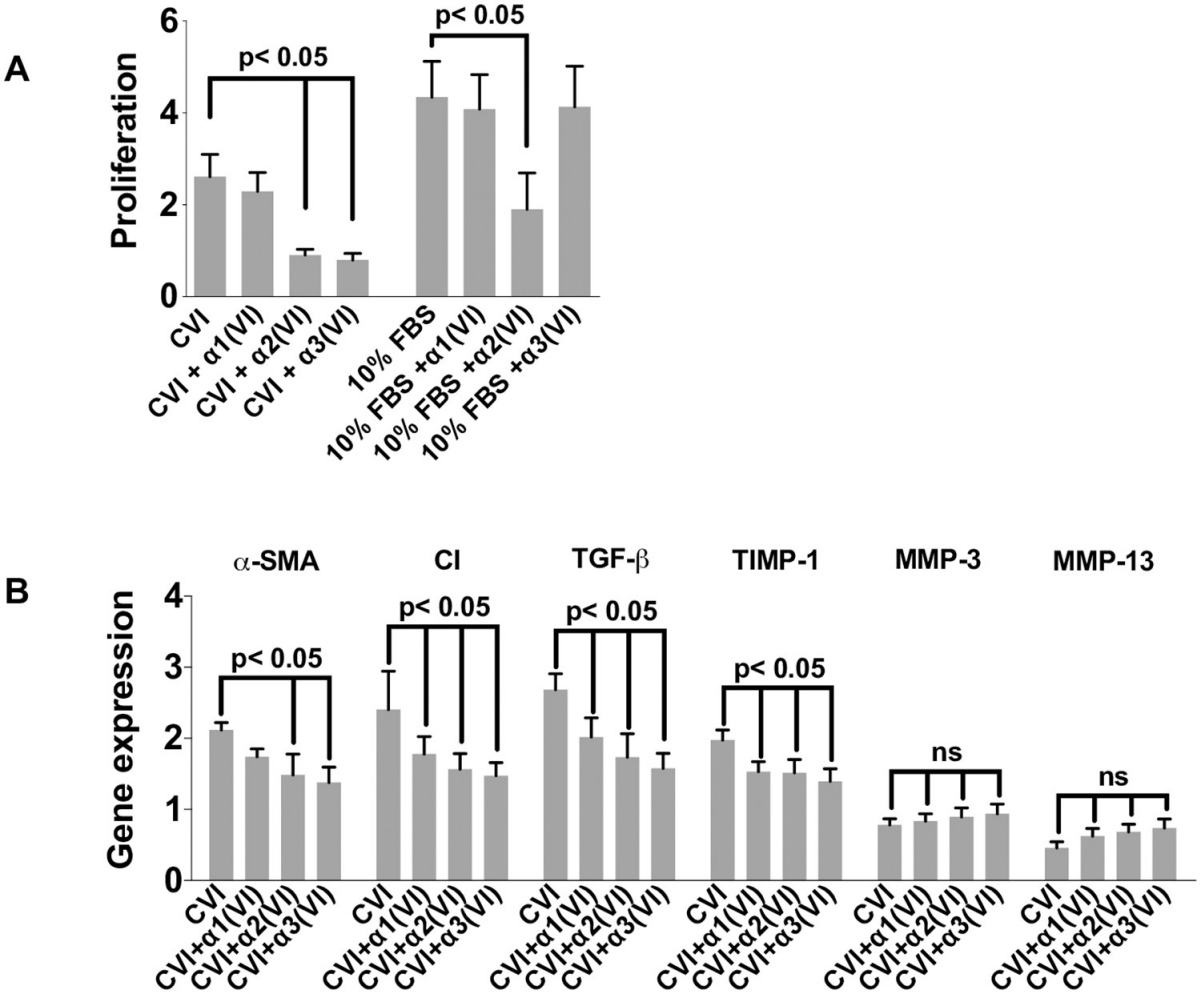
Effects of alpha single chains of CVI on 10% FBS- and CVI-induced proliferation and CVI-induced expression of fibrosis-associated genes in HSC. (**A**) Serum-starved CFSC were treated as indicated for 24 h. Proliferation was determined by [^3^H]-thymidine incorporation. Shown are Mean ± SD (n = 4). (**B**) Serum-starved CFSC were treated as indicated for 24 h. Gene expressions were determined by quantitative PCR and normalized to GAPDH. Shown are mean values ± SD (n = 4). Fig 4 Statistics were calculated by one-way ANOVA analysis and Tukey’s multiple comparisons test. Differences p<0.05 were considered significant.

The use of competitive fragments of ECM components has been proposed as potential therapeutic intervention to disrupt pro-fibrogenic ECM-cell interactions [22,33]. To test this for CVI, we isolated the three monomeric chains of CVI, α1–3 (VI) and assessed their impact on proliferation in primary rHSCs individually in the presence or absence of CVI. While the α2(VI) and α3(VI) chains inhibited DNA synthesis by 60% and 70% respectively, the α1(VI) chain did not show any effect, supporting the notion that the individual chains have different functional properties (Fig 4A; n = three independent experiments; p<0.05). To test the possibility that the single chains inhibited cell growth independent of CVI, we tested the effect of the individual chains on cell proliferation in rHSCs grown in 10% FBS. Interestingly, of the two inhibitory CVI chains, the α2(VI) also inhibited 10% FBS-induced proliferation, while the α3(VI) chain did not block any FBS-induced proliferation, indicating a specific, CVI-dependent effect of the α3(VI) chain (Fig 4A). Furthermore, the growth inhibitory effects of the α2(VI) and α3(VI) were not secondary to cytotoxic effects as shown by Calcein AM staining (S4 Fig). In contrast, the α1(VI) chain had no effect on CVI- or 10% FBS-induced proliferation (Fig 4A). These divergent impacts of the single chains were also apparent at the gene expression level and paralleled the results from the proliferation studies: Both, the α2(VI) or α3(VI), chains largely blocked the CVI-induced expression of fibrosis-associated genes including αSMA, CI and TGF-β, although the α1(VI) showed some inhibitory effects, albeit less pronounced (Fig 4B). All three single chains also significantly reduced the CVI-induced gene expression of TIMP-1. In contrast, the chains showed a trend towards reversing the downregulation of MMP-3 and MMP-13 expression although this was not statistically significant (Fig 4B).

**Fig 5:**
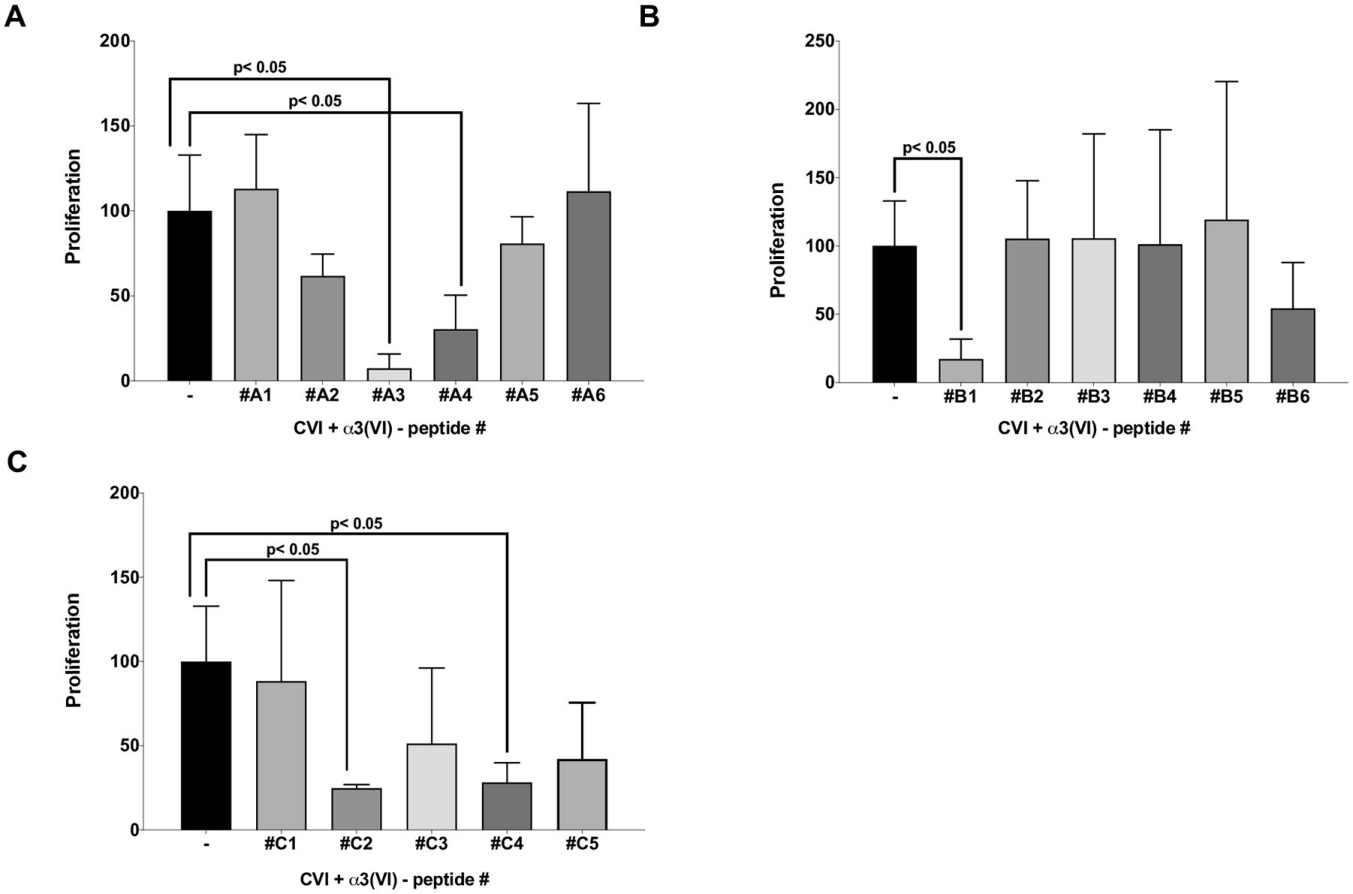
α3(VI)-derived peptides inhibit soluble CVI-induced HSC proliferation. (A-C) Serum-starved CFSC were cultured for 24 h with CVI in the presence or absence of the indicated synthetic 30-mer peptides derived from α3(VI) chains. Proliferation was determined by [^3^H]-thymidine incorporation. Shown are Mean ± SD (n = four independent experiments). Statistics were calculated by one-way ANOVA analysis and Tukey’s multiple comparisons test. Differences p<0.05 were considered significant.

To identify biologically relevant structural elements within the monomeric α3(VI)-chains, we generated a series of α3(VI)-derived peptides with a length of 30 amino acids (S1 Table). We focused on the α3(VI)-chain given that it specifically inhibited CVI effects and generated a total of 17 peptides and tested each of them individually as CVI competitors in proliferation studies. Of the 17 peptides tested, 9 did not show any inhibitory effect. However, while most of the peptides did not alter CVI-induced proliferation in CFSC, strong growth inhibitory effects were observed for the peptides A3, A4, B1, C2 and C4 whereas only A3, A4, B1 and C2, C4 significantly blocked CVI-induced proliferation (Fig 5; n = 4; p<0.05).

## Discussion

Here we have investigated the effects of soluble CVI and CVI-derived alpha single chains and synthetic peptides on survival, proliferation and fibrogenic phenotype in primary rat HSCs. Activated HSCs represent the major ECM-producing cell type [34,35] and, among other factors, upregulates CVI by 10-fold [8,9], which can be found on the cell surface of these cells and thus could function as a potent survival factor in vivo in line with the herein presented in vitro data. The functional importance of CVI for cell survival *in vivo* is further suggested by the significant levels of apoptosis in proximal muscle groups in humans and mice carrying a loss-of function mutation in CVI [36].

While the mechanisms underlying this anti-apoptotic property of CVI remain to be fully established, our studies point to a potent repression of BAX by soluble CVI. Bax, a pro- apoptotic member of the BCL-2 family, has been shown to be regulated by p53, which is up-regulated following serum withdrawal. This growth factor withdrawal-induced p53 and Bax upregulation can be mitigated by ECM components through integrin-mediated adhesion and spreading as shown for rat HSCs [37]. Indeed, blocking integrins with the pentapeptide Gly-Arg-Gly-Asp-Ser (GRGDS) has been shown to induce the expression of p53 and lead to elevated Bax levels and a 3-fold increase in the Bax/Bcl-2 ratio suggesting that integrins play a major role in Bax regulation through p53 [37].

Our studies demonstrate that the potent anti-apoptotic CVI properties are accompanied by enhanced HSC proliferation and significantly increased expression of fibrosis-associated genes. The increased deposition of CVI by HSCs could be an important auto- and paracrine mechanism aimed at extending the pool of activated HSCs. Such regulation could ultimately lead to rapid closure of the tissue damage via increased myofibroblast proliferation and deposition of ECM molecules [7]. This possibility is supported by the high circulating levels of CVI-fragments found in adults and children with advanced liver fibrosis [9,26]. Such a model would also open the opportunity to therapeutically modulate the CVI—HSC interaction using monomeric alpha single chains as competitors. Based on our previous findings that a mixture of the three CVI chains inhibit the mitogenic and anti-apoptotic effects of CVI in cell lines [20,30], we have now tested the potential of individual CVI-derived alpha single chains to inhibit CVI. Interestingly, the individual single chains have divergent effects: Of the three chains, the α2(VI) and α3(VI) inhibited the proliferative effects of CVI, although the α2(VI) chain also decreased FBS-induced proliferation suggesting that α2(VI) inhibits proliferation in a manner independent of CVI. In contrast, the effects of the α3(VI) chain are highly CVI dependent and suggest a specific interaction between CVI and the α3(VI) chain although the effect was studied at a 100-fold molar excess and the efficiency of lower doses has to be established in future studies. The α1(VI) showed no inhibitory effects. Furthermore, the α2(VI) and α3(VI) chains inhibited the expression of fibrosis-associated genes significantly stronger than α1(VI), indicating that different domains of the CVI molecule engage divergent signaling pathways. Structurally, this divergence is mirrored by the low similarity sequences in the X/Y positions, showing only 10–15% identical residues among α1(VI), α2(VI), and α3(VI) chains [38]. Such divergent effects between intact molecules and their subunits have been also described for other ECM components including endostatin that functions as a strong mitogen for many cell types in its oligomeric form while the monomers antagonize this effect [39].

Importantly, we have further narrowed down the functionally relevant domains of the α3(VI) chain through the use of 30 amino acid-long peptides and we identified several structural elements with inhibitory activity against mitogenic effects of CVI. Out of the three peptides with significant inhibitory activity, only the peptide C2 contains a typical integrin binding amino acid sequence (GKKGER) [40]. Other peptides with related amino acid sequences such as A5+A6 (GEKGER), A2 (GGPGER) and B5-B6 (GAPGER) showed no or weaker inhibitory activities. Of note, the integrin-binding motif RGD [41] was not found in the biologically active peptides. In conclusion, our studies suggest a model for the role of CVI in hepatic wound healing and fibrogenesis. During fibrogenesis, CVI degrading enzymes (such as MMPs or serine proteases) derived from inflammatory cells and HSC/myofibroblasts release soluble CVI fragments which act on HSC via its receptor-complex, which may contain integrins and the NG2 proteoglycan [21,42–44]. The exact composition of the receptor complex and entailed activation of intracellular signals need to be investigated in the future. Since the effects of CVI can be blocked by the monomeric alpha single chains of triple helical CVI as well as by α3(VI)-derived peptides, our results open the possibility of an ECM-based antifibrotic therapy.

## Supporting information

S1 Fig Soluble CVI regains its biological activity following denaturation and refolding CVI was denaturated and refolded by dialysis and gel filtration as described in the methods section. The proliferative effect of the reconstituted and non-reconstituted CVI at 100mg/ml on CFSC was determined through a [^3^H]-thymidine incorporation assay. Shown are Mean ± SD of three independent experiments. Statistics were calculated by one-way ANOVA analysis and Tukey’s multiple comparisons test. Differences p<0.05 were considered significant.
(PDF)

S2 Fig Collagen CVI promotes spreading and cytoplasmatic extension Primary HSC were plated at a density of 5x10^5^ HSC in Lab-Tek^™^ chamber-slides. After 24 h, cells were treated for five days with soluble CI, intact CVI or soluble CVI or with standard medium containing 10% FBS. 24 h after plating actin were stained with Phalloidin-TRITC. Arrows: round-shaped HSC, arrowheads point to cytoplasmic extensions.
(PDF)

S3 Fig Soluble CVI and TGF-β induce comparable gene expressions changes in HSCs Time-dependent effects of soluble CVI and TGF-β on CI expression was determined in CFSC cells. CFSC cells were cultured in medium containing 0.2% FBS with or without supplementation of soluble CVI or TGF-β for 24 h. Gene expression changes were assessed by quantitative PCR and normalized to GAPDH at indicated time points. Shown are representative data from one out of two independent experiments. Statistics were calculated by two-way ANOVA and Sidak’s multiple comparisons test and p< 0.05 was considered statistically significant.
(PDF)

S4 Fig Single CVI chains are not toxic To exclude that the effects of the alpha chains on proliferation or gene expression are due to cytotoxic effects, cells were treated 24 h with individual chains and cell viability was determined using the two-colour fluorescence Live/Dead^™^ Viability/Cytotoxicity Kit (Invitrogen^™^). Methanol (10% in medium) was used a positive control to induce cell toxicity. Medium was used as a non-toxic negative control. Since acetic acid (HAc) is used as solvent of the chains, cells treated with 0.15 M HAc were included as an additional control group. Shown are Mean ± SD of three independent experiments. Statistics were calculated by one-way ANOVA analysis and Tukey’s multiple comparisons test. Differences p<0.05 were considered significant.
(PDF)

S1 Table Amino acid sequences of α3(VI)-derived peptides. (PDF)

S2 Table Probes and primers for quantitative real-time PCR targeting rat cDNA. (PDF)

S1 Raw images Supplemental Western blot Raw Image Fig 1E. Supplemental Western blot Raw Image Fig 3D.
(PDF)
