# Supplementary figures and images for: Alpha-single chains of collagen type VI inhibit the fibrogenic effects of triple helical collagen VI in hepatic stellate cells
Source: PLoS One. 2021 Sep 2;16(9):e0254557. doi: 10.1371/journal.pone.0254557 (PMC8412337; doi:10.1371/journal.pone.0254557)

## Supporting information

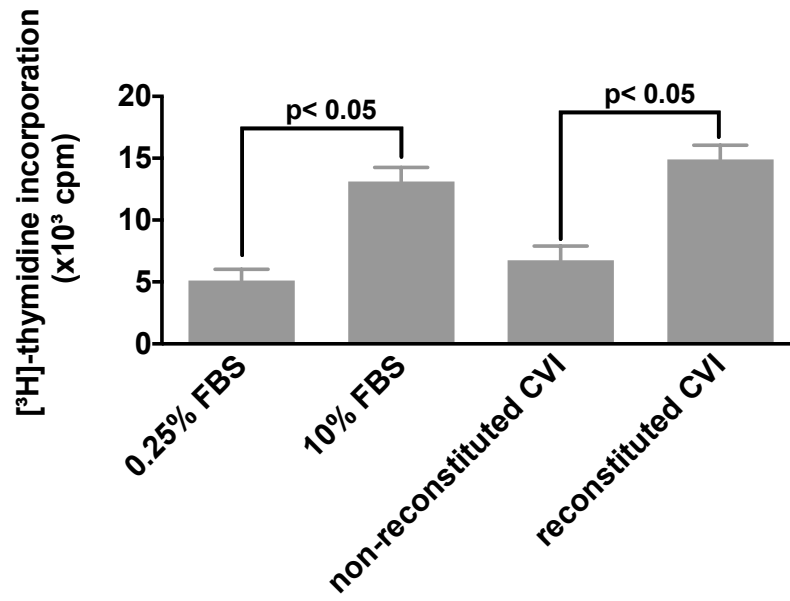

Figure S1

Supplement: S1 Fig — The proliferative effect of the reconstituted and non-reconstituted CVI at 100mg/ml on CFSC was determined through a [3H]-thymidine incorporation assay. Shown are Mean ± SD of three independent experiments. Statistics were calculated by one-way ANOVA analysis and Tukey’s multiple comparisons test. Differences p<0.05 were considered significant. (PDF) [file pone.0254557.s001.pdf]

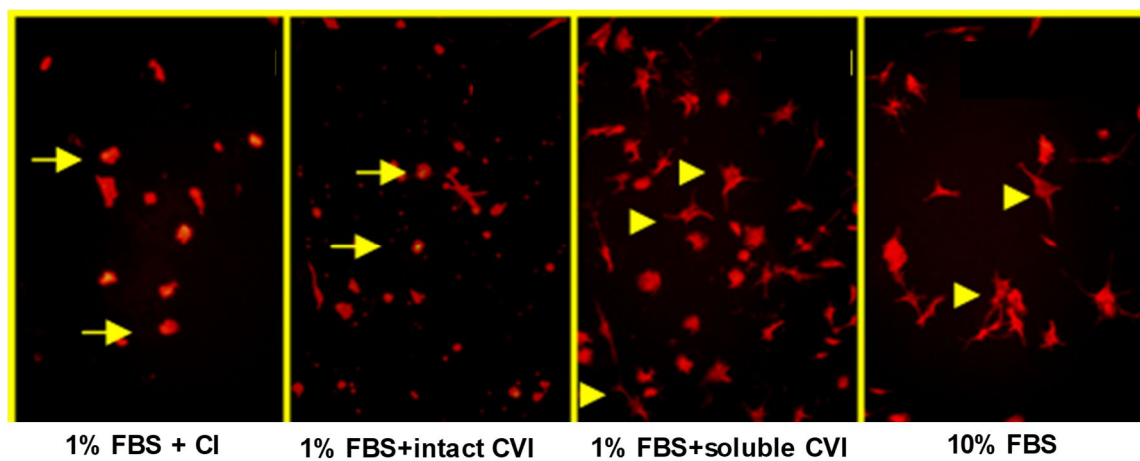

**Figure S2**

Supplement: S2 Fig — After 24 h, cells were treated for five days with soluble CI, intact CVI or soluble CVI or with standard medium containing 10% FBS. 24 h after plating actin were stained with Phalloidin-TRITC. Arrows: round-shaped HSC, arrowheads point to cytoplasmic extensions. (PDF) [file pone.0254557.s002.pdf]

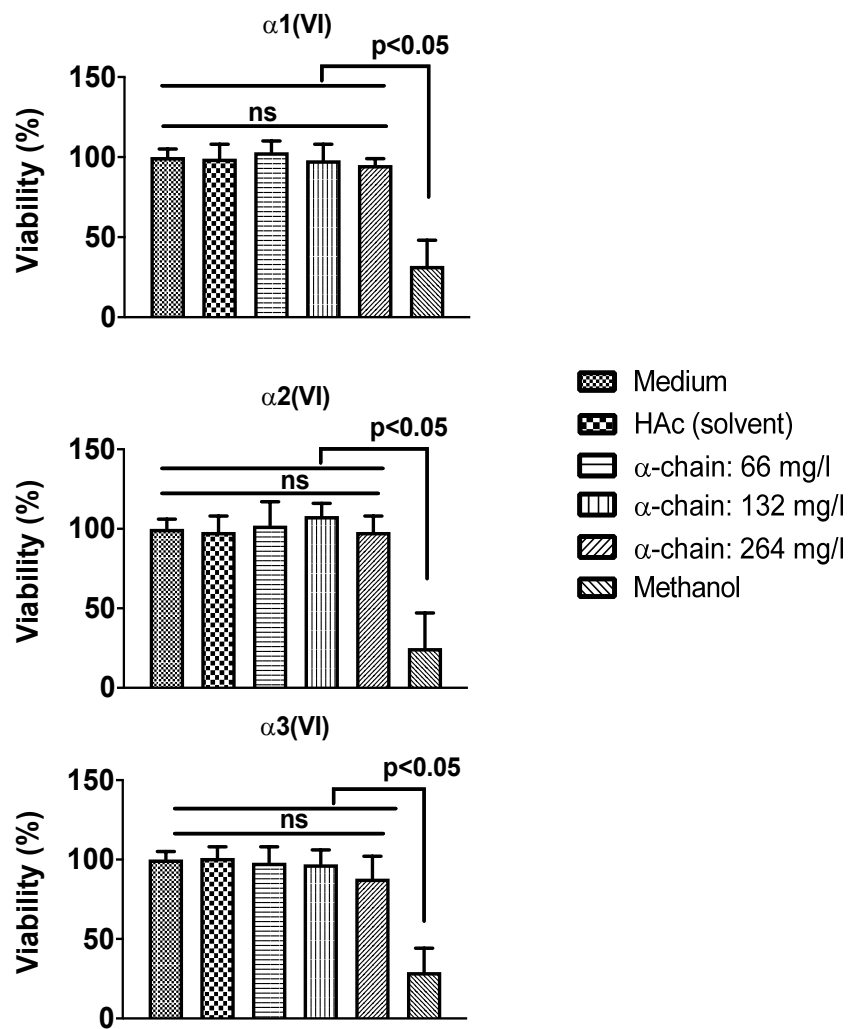

**Figure S4**

Supplement: S4 Fig — Methanol (10% in medium) was used a positive control to induce cell toxicity. Medium was used as a non-toxic negative control. Since acetic acid (HAc) is used as solvent of the chains, cells treated with 0.15 M HAc were included as an additional control group. Shown are Mean ± SD of three independent experiments. Statistics were calculated by one-way ANOVA analysis and Tukey’s multiple comparisons test. Differences p<0.05 were considered significant. (PDF) [file pone.0254557.s004.pdf]

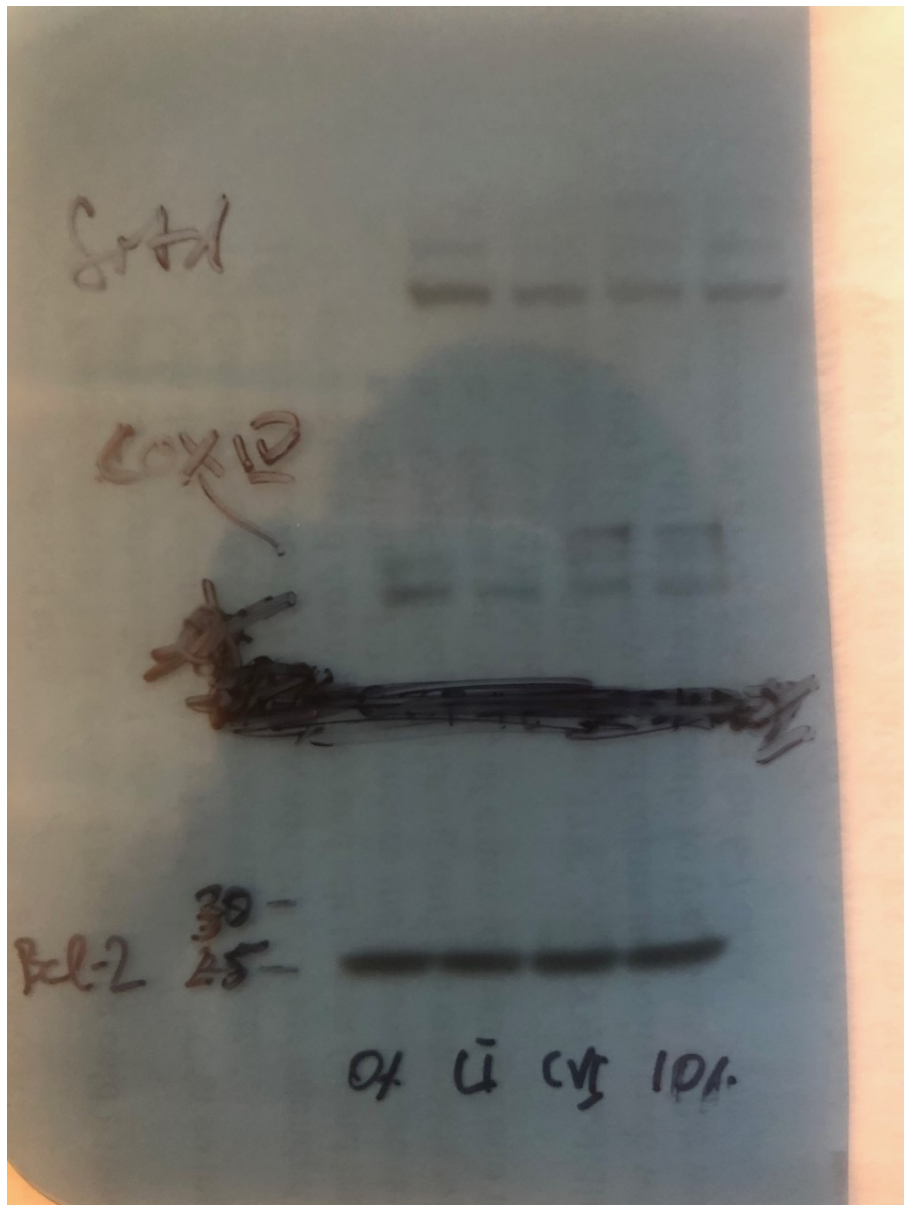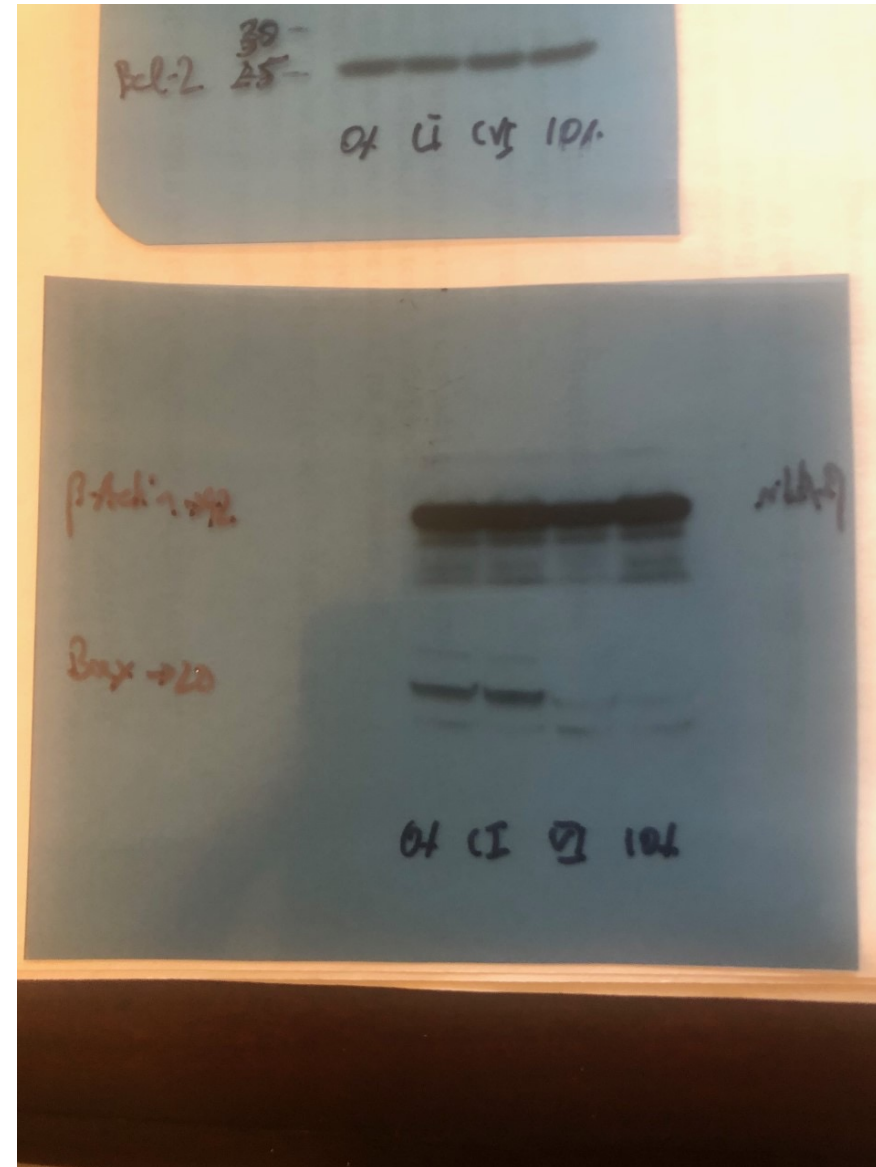

Western blot Raw Image  
Fig. 1E

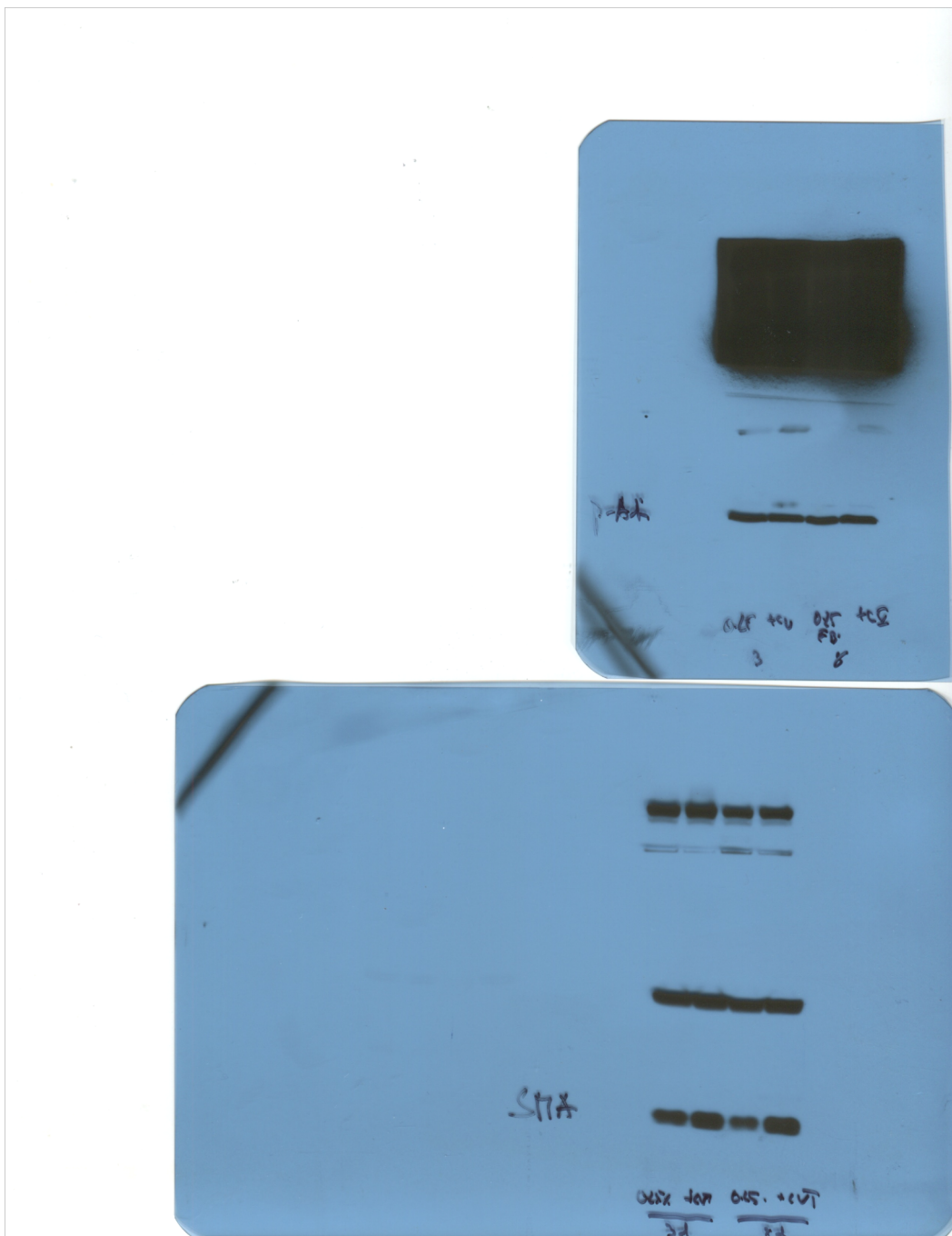

Western blot Raw Image Fig. 3D

Supplement: S1 Raw images — Supplemental Western blot Raw Image Fig 3D. (PDF) [file pone.0254557.s007.pdf]
