# Supplementary material for: Alpha-single chains of collagen type VI inhibit the fibrogenic effects of triple helical collagen VI in hepatic stellate cells
Source: PLoS One. 2021 Sep 2;16(9):e0254557. doi: 10.1371/journal.pone.0254557 (PMC8412337; doi:10.1371/journal.pone.0254557)
Supplement: S3 Fig — CFSC cells were cultured in medium containing 0.2% FBS with or without supplementation of soluble CVI or TGF-β for 24 h. Gene expression changes were assessed by quantitative PCR and normalized to GAPDH at indicated time points. Shown are representative data from one out of two independent experiments. Statistics were calculated by two-way ANOVA and Sidak’s multiple comparisons test and p< 0.05 was considered statistically significant. (PDF) [file pone.0254557.s003.pdf]

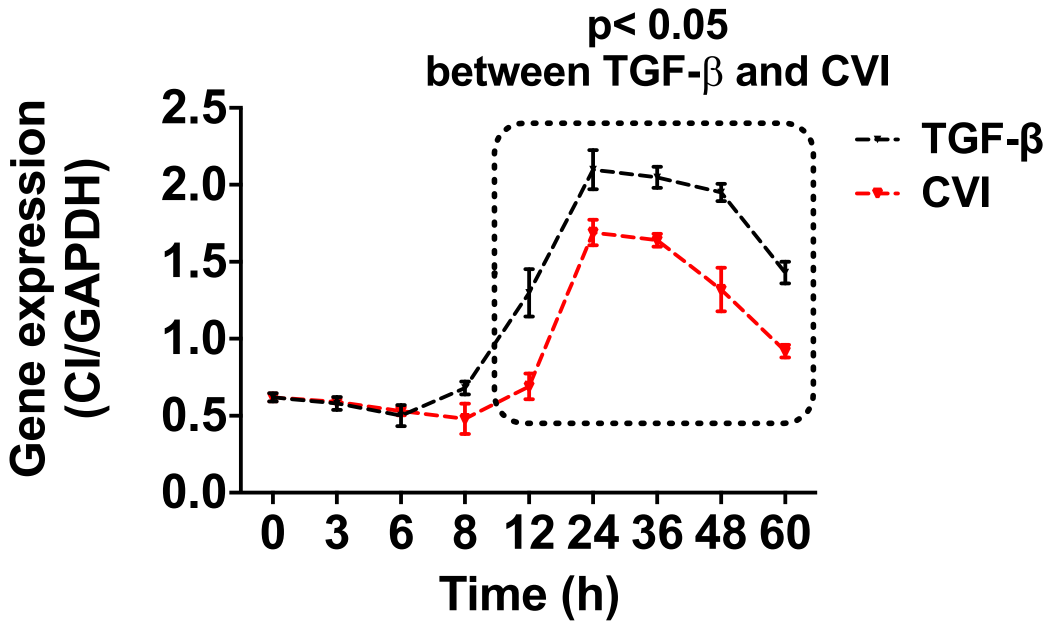
p< 0.05
between TGF-β and CVI
Gene expression
(CI/GAPDH)
2.5
2.0
1.5
1.0
0.5
0.0
0
3
6
8
12
24
36
48
60
Time (h)
TGF-β
CVI

Figure S3

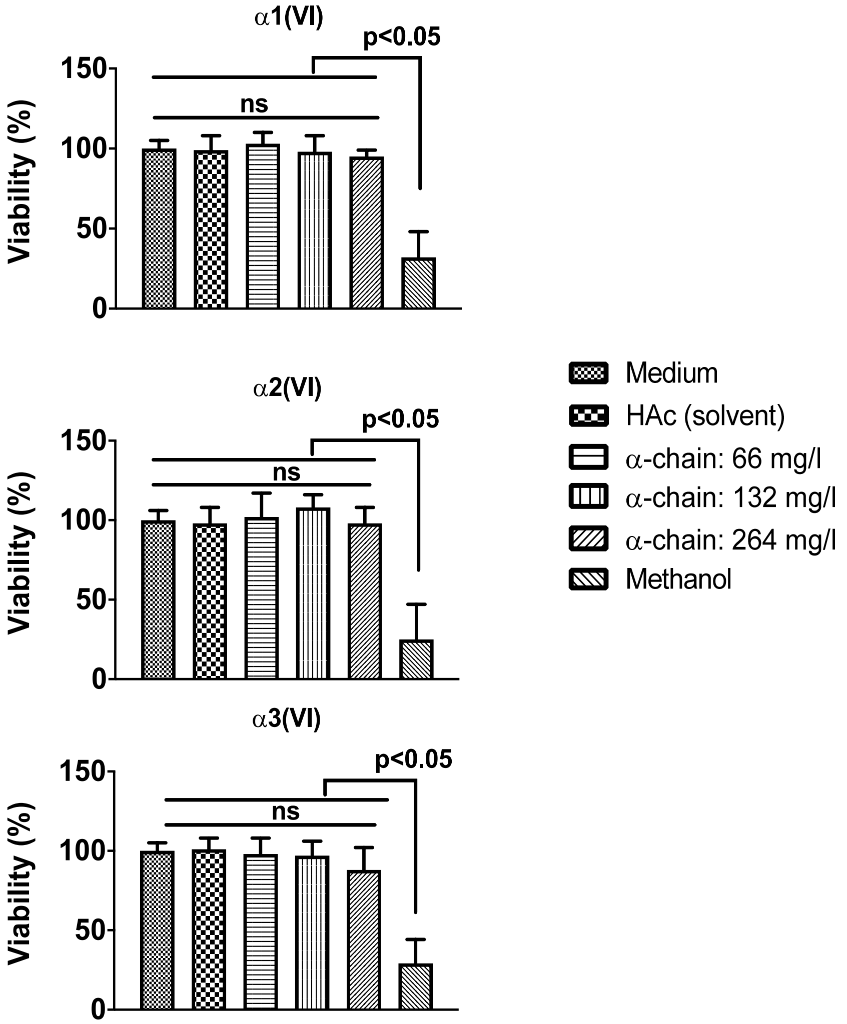
α1(VI)
p<0.05
ns
Viability (%)
150
100
50
0
α2(VI)
p<0.05
ns
Viability (%)
150
100
50
0
α3(VI)
p<0.05
ns
Viability (%)
150
100
50
0
Medium
HAc (solvent)
α-chain: 66 mg/l
α-chain: 132 mg/l
α-chain: 264 mg/l
Methanol

Figure S4

Table S1. Amino acid sequences of α3(VI)-derived peptides

NAME	PEPTIDE SEQUENCE
**A1**	GQRGDRGPIGSIGPKGIPGEDGYRGYPGDE
**A2**	DGYRGYPGDEGGPGERGPPGVNGTQGFQGC
**A3**	VNGTQGFQGCPGQRGVKGSRGFPGEKGEVG
**A4**	GFPGEKGEVGEIGLDGLDGEDGDKGLPGSS
**A5**	DGDKGLPGSSGEKGNPGRRGDKGPRGEKGE
**A6**	DKGPRGEKGERGDVGIRGDPGNPGQDSQER
**B1**	GNPGQDSQERGPKGETGDLGPMGVPGRDGV
**B2**	PMGVPGRDGVPGGPGETGKNGGFGRRGPPG
**B3**	GGFGRRGPPGAKGNKGGPGQPGFEGEQGTR
**B4**	PGFEGEQGTRGAQGPAGPAGPPGLIGEQGI
**B5**	PPGLIGEQGISGPRGSGGARGAPGERGRTG
**B6**	GAPGERGRTGPLGRKGEPGEPGPKGGIGNP
**C1**	PGPKGGIGNPGPRGETGDDGRDGVGSEGRR
**C2**	RDGVGSEGRRGKKGERGFPGYPGPKGNPGE
**C3**	YPGPKGNPGEPGLNGTTGPKGIRGRRGNSG
**C4**	GIRGRRGNSGPPGIVGQKGRPGYPGPAGPR
**C5**	GNSGPPGIVGQKGRPGYPGPAGPRGNRGDS

Table S2. Probes and primers for quantitative real-time PCR targeting rat cDNA

**Target**	**Oligonucteotide sequence (5′– 3′)**		
	**Probe[a]**	**Primer sense**	**Primer antisense**
CI	TTCTTGGCCATGCGTCAG GAGGG	TCCGGCTCCTGCTCCTCT TA	GTATGCAGCTGACTTCAGGG ATGT
TGF-β1	ACCGCAACAACGCAATCT ATGACAAAACCA	AGAAGTCACCCGCGTGCT AA	TCCCGAATGTCTGACGTATTG A
TIMP-1	TTCTGCAACTCGGACCTG GTTATAAGG	TCCTCTTGTTGCTATCATT GATAGCTT	CGCTGGTATAAGGTGGTCTC GAT
α-SMA	CTCGGCCGCCTGCTTCAC CA	CCTGCCAAGTATGATGAC ATCAAGA	GTAGCCCAGGATGCCCTTTA GT
MMP-3	AGATGGTATTCAATCCCTC TATGGACCTCC	CCGTTTCCATCTCTCTCAA GATGA	CAGAGAGTTAGATTTGGTGG GTACCA
MMP-13	TCTGGTTAGCATCATCATA ACTCCACACGT	GGAAGACCCTCTTCTTCT CA	TCATAGACAGCATCTACTTTG TC
GAPDH	TGGTGAAGCAGGCGGCC GAG	CCTGCCAAGTATGATGAC ATCAAGA	GTAGCCCAGGATGCCCTTTA GT
[a] Probes were labeled were labeled 5′ with 6-carboxy-fluorescein and 3′ with 6-carboxy-tetramethylrhodamin			
