# Supplementary material for: Alpha-single chains of collagen type VI inhibit the fibrogenic effects of triple helical collagen VI in hepatic stellate cells
Source: PLoS One. 2021 Sep 2;16(9):e0254557. doi: 10.1371/journal.pone.0254557 (PMC8412337; doi:10.1371/journal.pone.0254557)
Supplement: S1 Table — (PDF) [file pone.0254557.s005.pdf]

Table S1. Amino acid sequences of α3(VI)-derived peptides

NAME	PEPTIDE SEQUENCE
**A1**	GQRGDRGPIGSIGPKGIPGEDGYRGYPGDE
**A2**	DGYRGYPGDEGGPGERGPPGVNGTQGFQGC
**A3**	VNGTQGFQGCPGQRGVKGSRGFPGEKGEVG
**A4**	GFPGEKGEVGEIGLDGLDGEDGDKGLPGSS
**A5**	DGDKGLPGSSGEKGNPGRRGDKGPRGEKGE
**A6**	DKGPRGEKGERGDVGIRGDPGNPGQDSQER
**B1**	GNPGQDSQERGPKGETGDLGPMGVPGRDGV
**B2**	PMGVPGRDGVPGGPGETGKNGGFGRRGPPG
**B3**	GGFGRRGPPGAKGNKGGPGQPGFEGEQGTR
**B4**	PGFEGEQGTRGAQGPAGPAGPPGLIGEQGI
**B5**	PPGLIGEQGISGPRGSGGARGAPGERGRTG
**B6**	GAPGERGRTGPLGRKGEPGEPGPKGGIGNP
**C1**	PGPKGGIGNPGPRGETGDDGRDGVGSEGRR
**C2**	RDGVGSEGRRGKKGERGFPGYPGPKGNPGE
**C3**	YPGPKGNPGEPGLNGTTGPKGIRGRRGNSG
**C4**	GIRGRRGNSGPPGIVGQKGRPGYPGPAGPR
**C5**	GNSGPPGIVGQKGRPGYPGPAGPRGNRGDS
