# Supplementary material for: Alpha-single chains of collagen type VI inhibit the fibrogenic effects of triple helical collagen VI in hepatic stellate cells
Source: PLoS One. 2021 Sep 2;16(9):e0254557. doi: 10.1371/journal.pone.0254557 (PMC8412337; doi:10.1371/journal.pone.0254557)
Supplement: S2 Table — (PDF) [file pone.0254557.s006.pdf]

Table S2. Probes and primers for quantitative real-time PCR targeting rat cDNA

Target	Oligonucteotide sequence (5′– 3′)		
	**Probe[a]**	**Primer sense**	**Primer antisense**
CI	TTCTTGGCCATGCGTCAG GAGGG	TCCGGCTCCTGCTCCTCT TA	GTATGCAGCTGACTTCAGGG ATGT
TGF-β1	ACCGCAACAACGCAATCT ATGACAAAACCA	AGAAGTCACCCGCGTGCT AA	TCCCGAATGTCTGACGTATTG A
TIMP-1	TTCTGCAACTCGGACCTG GTTATAAGG	TCCTCTTGTTGCTATCATT GATAGCTT	CGCTGGTATAAGGTGGTCTC GAT
α-SMA	CTCGGCCGCCTGCTTCAC CA	CCTGCCAAGTATGATGAC ATCAAGA	GTAGCCCAGGATGCCCTTTA GT
MMP-3	AGATGGTATTCAATCCCTC TATGGACCTCC	CCGTTTCCATCTCTCTCAA GATGA	CAGAGAGTTAGATTTGGTGG GTACCA
MMP-13	TCTGGTTAGCATCATCATA ACTCCACACGT	GGAAGACCCTCTTCTTCT CA	TCATAGACAGCATCTACTTTG TC
GAPDH	TGGTGAAGCAGGCGGCC GAG	CCTGCCAAGTATGATGAC ATCAAGA	GTAGCCCAGGATGCCCTTTA GT

[a] Probes were labeled were labeled 5′ with 6-carboxy-fluorescein and 3′ with 6-carboxy-tetramethylrhodamin
